# Current practices and challenges in assisted reproductive technology care pathways in France and Belgium: the AMPLITUDE survey

**DOI:** 10.3389/frph.2025.1617628

**Published:** 2025-09-30

**Authors:** Christine Wyns, Christophe Blockeel, Anne Guivarc’h-Lévêque, Géraldine Porcu-Buisson, Nelly Swierkowski-Blanchard, Chadi Yazbeck, Catherine Rongières

**Affiliations:** ^1^ Department of Gynecology-Andrology, Cliniques Universitaires Saint-Luc, Brussels, Belgium; ^2^ Brussels IVF, Center for Reproductive Medicine Universitair Ziekenhuis Brussel, Vrije Universiteit Brussel, Brussels, Belgium; ^3^ Service of Reproductive Medicine, Clinique Mutualiste La Sagesse, Rennes, France; ^4^ Department of Reproductive Medicine, Institut de Médecine de la Reproduction, Marseille, France; ^5^ Reproductive Medecine Center, Intercommunal Hospital Center, Poissy, France; ^6^ Laboratory of Developmental Biology and Reproduction, RHuMA-TEAM, UMR-BREED, UFR-SVS, UVSQ, Montigny-le-Bretonneux, France; ^7^ Obstetrics Gynecology and Reproductive Medicine, Reprogynes Medical Institute, Paris, France; ^8^ Obstetrics Gynecology and Reproductive Medicine, Groupe Hospitalier Privé Ambroise Paré Hartmann, Neuilly-sur-Seine, France; ^9^ Department of Reproductive Medicine, Strasbourg University Hospital, Strasbourg, France

**Keywords:** ART, medically assisted reproduction, care pathway, patients expectations, patients support, barriers to care, quality of life, survey

## Abstract

**Introduction:**

This study aimed to evaluate current practices in assisted reproductive technology (ART) patient care, identifying potential areas for improvement. Collective data will further provide key insights in gaps and potential new tools to enhance ART care practices for patients and healthcare professionals.

**Methods:**

An online questionnaire comprising 22 multiple choice questions was distributed to ART specialists in France and Belgium between September and November 2023. Responses were analyzed overall and by country. Descriptive analysis used 5-point Likert scales (converted to numerical scores) for comparative insights. Qualitative data were reported as frequencies (%), and quantitative data as means and standard deviations.

**Results:**

A total of 166 IVF specialists participated in the survey out of 487 contacted, 130 from France (78.3%) and 36 from Belgium (21.7%). Most respondents (92.8%) scheduled the first consultation within three months, with all Belgian specialists meeting this timespan compared to 90.8% in France. Notably, 30.8% of French specialists and 29.5% of Belgian specialists scheduled appointments within one month. During initial consultations, 73.3% provided patients with informational materials, and 61.5% informed them about psychological support options. To assess lifestyle factors, clinicians primarily used oral questionnaires (91.9%), with a higher prevalence of written questionnaires in Belgium compared to France (37.1% vs. 15.9%). When patients struggled to understand treatment instructions, 82.6% of clinicians took time to re-explain, and 60.9% referred patients to nursing staff for further assistance. Most respondents (90.7%) provided digital tools for injection training, while 74.7% offered training sessions conducted by paramedical staff. Most reported treatment errors included dosage and handling mistakes and nurse injection errors. Psychological support was offered by 80% of respondents for IVF failures, with variations between countries in follow-up approaches. Overall, clinicians rated patients’ knowledge of different ART aspects as relatively low, with average scores ranging from 2.43/5 to 3.30/5, depending on the items.

**Discussion:**

The main areas for improvement highlighted in this study were patient education and support throughout the care pathway. Differences in practices between France and Belgium were also observed, highlighting the importance of context-adapted approaches. Our observations may further facilitate the development of tailored tools aimed at improving ART care practice.

## 1 Introduction

The Assisted Reproductive Technology (ART) healthcare pathway is associated with a relatively low birth rate, approximately 25% (1). Consequently, the process can be particularly challenging for patients, with significant emotional (stress, anxiety, depression), physical (pain, side effects from treatment), and occupational impacts (due to the need for frequent consultations, daily treatments) (2). A meta-analysis involving 10,000 infertile women showed that 4 out of 10 patients suffer from depression (3). Additionally, patients' hope for a positive outcome significantly diminishes after multiple failed attempts: the psychological burden of treatment and repeated failures are key contributors to the high dropout rate (4, 5).

Beyond the physical and psychological burden, the fertility journey is also highly complex. According to a study conducted in 28 ART centers in France, only 58% of patients reported understanding the treatment instructions given by their gynecologist. This misunderstanding often leads to treatment errors, particularly during ovarian stimulation, thus hindering the proper execution of ART protocols. Differences between the prescribed treatment and patients' understanding were observed in 21% of cases (2).

Besides that, infertility is on the rise, currently affecting approximately 1 in 6 couples globally, according to the World Health Organization (WHO). As a result, ART centers are facing an increasing influx of patients. This surge in activity has been further intensified in recent years due to expanding indications for ART. These new indications include fertility preservation cycles for medical or social reasons, as well as ART access for diverse family compositions including same-sex couples and single individuals.

In a context where patients are growing in numbers, but also more demanding, healthcare professionals must adapt their practices to meet these evolving behaviors and optimize the ART care pathway. To identify potential areas for improvement, the objective of this practice survey was to provide a snapshot and assessment of how patients undergoing ART are currently being supported by healthcare professionals. While several of the challenges analyzed in this study, such as patient anxiety and need for better education have already been reported, we aimed at covering literature gaps on the level of patient knowledge and variations in support practices. As these issues are likely to exist in many countries, our findings may serve as a reference point or a cautionary example for other healthcare systems seeking to improve ART care.

## 2 Methods

### 2.1 Drawing up the questionnaire

The questionnaire was developed by a committee of seven ART experts, leaders in Reproductive Medicine (five from France and two from Belgium), based on a literature review and their clinical experience. Its content and wording were finalized and validated by consensus during two dedicated meetings to ensure clarity, relevance and neutrality. Particular care was taken to ensure that these experts came from different regions of France (Bretagne, Grand-Est, Ile-de-France, Provence-Alpes-Côte d'Azur) and Belgium (Brussels area). It consisted of 22 closed questions divided into 5 sections: profile of the respondent, data on the first appointment, support at initiation and during treatment, managing failure, and trends in ART (patients' understanding of administrative procedures, access prerequisites, reimbursement conditions, and patient rights). It also examined obstacles encountered in daily practice and adaptations implemented following the new applications of French Bioethics Law. Given that the survey was conducted in two different contexts, certain items were not proposed to the Belgian respondents for this last section (new applications following 2021 Bioethics Law and No preimplantation diagnostics). Indeed, there are notable differences in the regulatory framework of ART between the two countries. France recently updated its Bioethics Law in 2021, introducing new applications for ART (Loi n°2021-1017 du 2 août relative à la bioéthique), while Belgium has had a more permissive approach to ART for several years. Additionally, preimplantation diagnosis regulated differently in these countries, with Belgium allowing it under broader circumstances compared to France.

### 2.2 Distribution of the questionnaire

The questionnaire was distributed online via a secure platform (Made In Surveys by MIS Group) between September 13, 2023, and November 13, 2023. During this period, non-respondents were followed up weekly by email. The detailed questionnaire is presented in Supplementary Material. This questionnaire was sent to 487 healthcare professionals treating patients undergoing ART (obstetric gynecologists, medical gynecologists, medical biologists, endocrinologists) and practicing IVF in France (*n* = 384) and Belgium (*n* = 103). Responding to the survey was volunteer, unpaid, and anonymous. Each participant was assigned a unique identifier linked to their email for reminders but separate from their survey responses. This ensured we could follow up with non-respondents while maintaining response anonymity.

### 2.3 Analysis

After distribution, an initial analysis was carried out for all respondents. A second sub-group analysis was then carried out for respondents from Belgium and France. The analysis was carried out by the Medical Education agency KPL, Paris, and the Scientific Committee validated the final results. Qualitative data were expressed as frequency (% of the population or sub-population considered) and quantitative data were expressed as mean and standard deviation. For some questions, a descriptive analysis was conducted using 5-point Likert scales, where each response level was converted into a numerical score (1–5), allowing for the calculation of means and enabling a synthetic and comparative analysis of the results. This approach provided a quantitative measure of tendency for responses, facilitating comparisons across different items.

## 3 Results

### 3.1 Profile of respondents

The questionnaire was sent to 487 IVF specialists (384 French and 103 Belgian). Overall, 166 IVF specialists completed the questionnaire and reached at least the second section, resulting in a response rate of 34.1%. The 487 specialists contacted represented nearly all prescribers for whom we had contact information. The 34.1% response rate covered more than 50% of the centers, with good geographical distribution across the territory, ensuring a satisfactory level of representativeness. Among the respondents, 130 practiced in France (78.3% of total respondents; 33.9% response rate among French specialists) and 36 in Belgium (21.7% of total respondents; 35.0% response rate among Belgian specialists). Of these respondents, 139 completed all the questions. The characteristics of the respondents are shown in Table 1. Partial responses were included in the analysis if the respondent had completed at least up to question 6.

**Table 1:** Characteristics of the respondents.

	All (*n* = 166)	France (*n* = 130)	Belgium (*n* = 36)
Medical speciality—*n* (%)			
Obstetric gynecologists	97 (58.4)	68 (52.3)	29 (80.6)
Medical gynecologists	42 (25.3)	35 (26.9)	7 (19.4)
Medical biologists	19 (11.6)	19 (14.6)	0 (0.0)
Endocrinologists	8 (4.9)	8 (6.2)	0 (0.0)
Practice—*n* (%)			
Hospital	77 (46.4)	54 (41.5)	23 (63.9)
Private	55 (33.1)	53 (40.8)	2 (5.6)
Both	34 (20.5)	23 (17.7)	11 (30.6)
Age—*n* (%)			
<30 years old	3 (1.3)	3 (2.3)	0 (0.0)
[31–40] years old	59 (35.5)	45 (34.6)	14 (38.9)
[41–50] years old	53 (31.9)	40 (30.8)	13 (36.1)
[51–60] years old	29 (17.5)	23 (17.7)	6 (16.7)
>60 years old	22 (13.3)	19 (14.6)	3 (8.3)
Location—*n* (%)			
Ile-de-France	27 (16.3)	27 (20.8)	–
Auvergne-Rhône-Alpes	21 (12.7)	21 (16.2)	–
Hauts-de-France	20 (12.0)	20 (15.4)	–
Grand-Est	16 (9.6)	16 (12.3)	–
Provence-Alpes-Côte d’Azur	11 (6.6)	11 (8.5)	–
Pays de la Loire	9 (5.4)	9 (6.9)	–
Bretagne	7 (4.2)	7 (5.4)	–
Nouvelle-Aquitaine	5 (3.0)	5 (3.8)	–
Occitanie	4 (2.4)	4 (3.1)	–
Bourgogne-Franche-Comté	4 (2.4)	4 (3.1)	–
Normandie	3 (1.8)	3 (2.3)	–
Centre-Val-de-Loire	3 (1.8)	3 (2.3)	–
Flanders	16 (9.6)	–	16 (44.4)
Wallonia	11 (6.6)	–	11 (30.6)
Brussels	9 (5.4)	–	9 (25.0)

### 3.2 The first appointment

**Figure 1:**
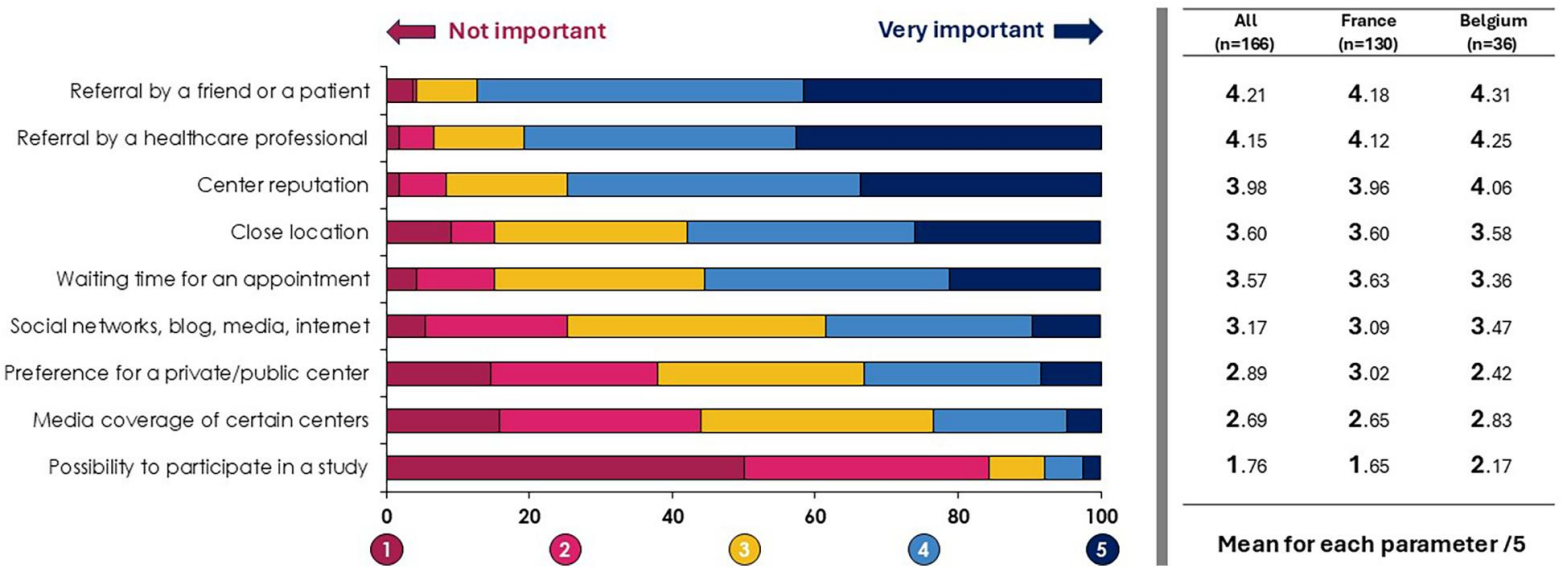
Factors influencing patients when selecting a fertility center.

The majority of respondents reported scheduling the first consultation within 3 months (154/166; 92.8%). This trend was observed in both Belgium (36/36; 100%) and France (118/130; 90.8%). A higher proportion of French respondents scheduled appointments within one month compared to their Belgian counterparts (30.8%, 40/130 vs. 25.0%, 9/36). This waiting time for an appointment was ranked 5th among all factors influencing patients when selecting a fertility center (Figure 1). As shown in Figure 1, referrals from friends, patients or healthcare professionals, as well as the center reputation and the center location were the most critical criteria. In Belgium, the influence of social networks, blog, media, and internet was rated as more important than the waiting time for an appointment, in patients' decision-making process. A minority of respondents reported not sharing any information about the treatment pathway during the initial consultation (24/166; 14.9%), with similar results in France (19/130; 15.1%) and in Belgium (5/36; 14.3%). Nearly three quarters of respondents (118/166; 73.3%) said they shared documentation with their patients, in a variety of formats (brochures, flyers, videos, etc.). These results were comparable among French and Belgian respondents (93/130; 73.8% and 25/36; 71.4%, respectively). Respondents also informed their patients about the possibility to meet a psychologist (99/166; 61.5%), to have informational meetings on therapeutic education (54/166; 33.5%), or to get in touch with patient associations (16/166; 9.9%). To assess patients' lifestyle and exposure to toxic factors, respondents primarily used an oral questionnaire (148/161; 91.9%), written questionnaire (33/161; 20.5%), and biological check-up (23/161; 14.3%). The use of written questionnaire appeared to be more widespread in Belgium, compared to France (13/36; 37.1% vs. 20/130; 15.9%).

### 3.3 Support at initiation and during treatment

When patients have difficulty understanding treatment instructions from prescribers, the latter adopted the following strategies: taking time to re-explain (133/161; 82.6%), referring the patient to a nurse or a midwife (98/161; 60.9%), asking the patient to bring a trusted person (76/161; 47.2%), or proposing teleconsultations (19/161; 11.8%). Six out of 10 respondents indicated they would use a translator if necessary (97/161; 60.2%).

To ensure injections are carried out correctly, the majority of respondents provided digital tools to empower patients (147/162; 90.7%). These results were similar in France (115/126; 91.3%) and in Belgium (32/36; 88.9%). Respondents from both countries also proposed injection training provided by the paramedical team (121/162; 74.7%). Nearly two-thirds of respondents preferred to demonstrate themselves how to perform injections (99/162; 61.1%). Finally, 19.1% of respondents proposed a program to support patient's pathway (31/162; 19.1%).

The frequence of treatments errors reported by clinicians was assessed using a 5-point Likert scale, with 5 being the highest error's frequency. The most common errors reported by clinicians were dosage and handling errors (2.46/5), nurse injection errors (2.31/5), wrong time of medication intake (2.13/5), and incorrect dispensing of medication (2.02/5). Other types of errors, including forgetting to take treatment, inverting different drugs, storage problems, and missing a follow-up appointment, seemed to be relatively less frequent (<2/5 average score).

### 3.4 Managing failure

We asked clinicians what kind of procedures have been implemented in their center to manage failures of IVF procedures. We differentiated between pre-transfer and post-transfer failures. Results are described in Table 2a. The main approach proposed to patients was psychological support (eight out of ten responders indicated offering psychological support to their patients). This approach was more frequent when failure occurred after embryo transfer (+5.0%). Additional check-up and alternative medicine workshops were also more often proposed when failure occurred after transfer (+20.3% and +10.0%, respectively). Table 2b describes the different approaches in France and Belgium.

**Table 2:** Management of IVF failure.

	Failure before embryo transfer		Failure after embryo transfer	
2a. Management strategies proposed before and after embryo transfer				
Strategies proposed to patients—*n* (%)				
Psychological support	111 (79.3)		118 (84.3)	
Systematic consultation	96 (68.6)		87 (62.1)	
Additional check-up	82 (58.3)		110 (78.6)	
Team trained to announce and manage failure	65 (47.1)		64 (45.7)	
Alternative medicine workshops proposed	39 (27.9)		53 (37.9)	
Participation in discussion groups	28 (20.0)		32 (22.9)	
Collaboration with patient associations	15 (10.7)		18 (12.9)	
2b. Comparison of management strategies in France and Belgium				
Strategies proposed to patients—*n* (%)	France	Belgium	France	Belgium
Psychological support	83 (78.3)	28 (82.4)	90 (84.9)	28 (82.4)
Systematic consultation	70 (66.0)	26 (76.5)	63 (59.4)	23 (67.6)
Additional check-up	58 (54.7)	24 (70.6)	84 (79.2)	26 (76.5)
Team trained to announce and manage failure	46 (43.3)	20 (58.8)	44 (41.5)	20 (58.8)
Alternative medicine workshops proposed	35 (33.0)	4 (11.8)	46 (43.4)	7 (20.6)
Participation in discussion groups	24 (22.6)	4 (11.8)	27 (25.5)	5 (14.7)
Collaboration with patient associations	11 (10.4)	4 (11.8)	13 (12.3)	5 (14.7)

### 3.5 Trends in ART

**Figure 2:**
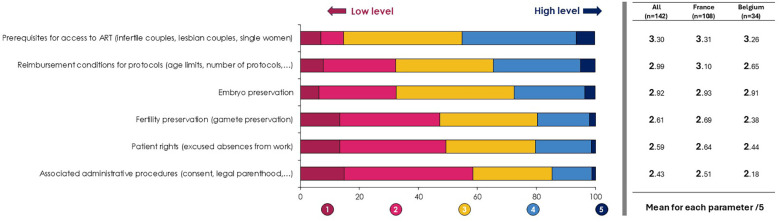
Level of patient knowledge of ART.

We proposed to evaluate patients' level of knowledge of ART, according to six items. The general level of knowledge was relatively low according to respondents, with an average score between 2.43/5 and 3.30/5. Results are described in Figure 2.

The different obstacles encountered by respondents in their daily practice are described in Table 3. Concerning the changes in French practices following the Bioethics Law revision, 41.1% of respondents indicated they did not change their practices. Others set up special appointments for new patient profiles (37.4%), recruited healthcare professionals (32.7%), recruited administrative staff (24.3%), implemented new follow-up tools (15.9%).

**Table 3:** Obstacles encountered by IVF specialists.

	All (*n* = 139)	France (*n* = 106)	Belgium (*n* = 33)
Obstacles—*n* (%)			
Lack of financial resources and equipment	78 (56.1)	57 (53.8)	21 (63.6)
Lack of paramedical staff	72 (51.8)	53 (50.0)	19 (57.6)
Very large number of active patients	62 (44.6)	51 (48.1)	11 (33.3)
Lack of medical staff	57 (41.0)	49 (46.2)	8 (24.2)
Waiting time for the first appointment	35 (25.2)	25 (23.6)	10 (30.3)
Waiting time between 1st appointment and treatment initiation	29 (20.9)	20 (18.9)	9 (27.3)
New applications following 2021 Bioethics law	–	63 (59.4)	–
No preimplantation diagnosis	–	52 (49.1)	–

## 4 Discussion

This survey provides valuable insights into the current practices and challenges in ART care in France and Belgium. The results highlight several key areas for discussion and the potential for improvement in patient care. However, it is important to note the study's limitations, including its reliance on clinician perceptions rather than direct patient feedback. Future research could benefit from incorporating patient perspectives to provide a more comprehensive view of the ART care pathway. It is important to acknowledge the possibility of selection bias, as respondents may be those more engaged or sensitive to these issues, and therefore, the practices described here may not fully reflect those of all ART clinics. Moreover, the findings rely on practitioners' perceptions, which may differ from patients' own experiences and perspectives. We can also mention a potential desirability bias (idealization and favorable presentation of responses by respondents), which can affect self-assessment (6).

### 4.1 Patient information and support

Our findings reveal that while most clinicians (85.1%) provide information during the initial consultation, the relatively low level of patient's knowledge reported by clinicians (average scores between 2.43/5 and 3.30/5) underscores the need for more effective communication strategies. This aligns with a previous study showing that only 58% of patients fully understand treatment instructions (2). This level of knowledge on the different aspects of an ART treatment pathway appears to be relatively the same in both countries. However, Belgian respondents consider their patients less familiar with the reimbursement conditions for protocols (2.65/5 vs. 3.10/5) and the associated administrative procedures (2.18/5 vs. 2.51/5), compared with the responses of French clinicians.

The high prevalence of digital tools (90.7%) and injection training (74.7%) provided by clinicians is encouraging. However, the frequency of dosage and handling errors (2.46/5) suggests these methods may not be fully effective. Interestingly, practices differ between countries: Belgian professionals rely more on their team to perform injection training while French respondents more often demonstrate the procedure themselves. Also, Belgian clinicians were more likely to offer therapeutic education sessions compared to their French counterparts. They also address the patient to a nurse/midwife more easily than in France, in case of trouble for understanding treatment instructions. France has virtually no restrictions on medication reimbursement, whereas Belgium's fixed-fee system forces practitioners to minimize waste, making ART teams more directly involved in patient education. Conversely, the French respondents make much more use of teleconsultation, which is totally absent from the practice of Belgian healthcare professionals. In France, teleconsultation grew with COVID-19, while in Belgium it remains rare, as reimbursement only started during COVID and patients live closer to ART centers. Moreover, none of the Belgian respondents mentioned the existence of patient associations to their patients, unlike the French where such associations appear more prominent and active, offering stronger support networks than in Belgium These results highlight the need to develop specific tools tailored to the organization of each IVF center and adapted to each country's system, in order to improve the quality of care.

Future research could explore more innovative approaches to patient education and support, potentially leveraging technology for personalized instruction and follow-up.

### 4.2 Managing treatment failure

The emphasis on psychological support following IVF failure (80% of respondents) is a positive finding, given the known emotional impact of unsuccessful treatments.

As a complement to this approach, measures set up differ between countries: team training in failure management is more frequent in Belgium, for example. Also, Belgians more often schedule additional appointments or check-ups. French respondents more frequently propose alternative medicine workshops and discussion groups after failure. Implementing more comprehensive support systems throughout the treatment process could potentially reduce dropout rates and improve patient well-being.

### 4.3 Healthcare professional challenges

The obstacles reported by clinicians, particularly in France following the revision of the Bioethics law, highlight the need to adapt human resource capacity and resize logistics in order to respond directly to changes in regulations and the influx of patients. The recruitment of additional staff and implementation of follow-up tools by some centers represent positive steps, but more widespread and standardized approaches may be beneficial.

### 4.4 Conclusion and future directions

While current ART care practices in France and Belgium show many strengths, there are clear opportunities for improvement. Enhancing patient education, providing consistent support throughout the treatment process, and addressing the challenges faced by healthcare professionals could possibly contribute to better outcomes and experiences for patients. Future research should incorporate patient perspectives to provide a more comprehensive view of the ART care pathway and explore innovative approaches to patient education and support, potentially leveraging technology for personalized instruction and follow-up. Beyond the French and Belgian contexts, these findings may have broader relevance, highlighting common challenges in ART care and emphasizing the need for globally applicable strategies to improve patient education, support, and overall care pathways.

This survey identified barriers in the ART care pathway and clinicians' expectations. Ongoing work with 38 healthcare professionals from France and Belgium focuses on developing tailored tools to address the issues raised in this study. Additionally, a Delphi consensus on ovarian stimulation management (7) provides further insights into optimizing clinical practices and could serve as a foundation for improving care strategies.

## Data Availability

The original contributions presented in the study are included in the article/Supplementary Material, further inquiries can be directed to the corresponding author.
