# Supplementary material for: Current practices and challenges in assisted reproductive technology care pathways in France and Belgium: the AMPLITUDE survey
Source: Front Reprod Health. 2025 Sep 30;7:1617628. doi: 10.3389/frph.2025.1617628 (PMC12518220; doi:10.3389/frph.2025.1617628)
Supplement: Supplementary file 1 [file Table1.docx]

Supplementary Material

**Supplementary Material.** Questionnaire distributed online

1. **YOUR PROFILE**

**Q1. How old are you?** *[only one choice possible]*

- ≤30 years
- Between 31 and 40 years
- Between 41 and 50 years
- Between 51 and 60 years
- >60 years

**Q2. What is your specialty?** *[only one choice possible]*

- Gynecologist-obstetrician
- Medical gynecologist
- Medical biologist
- Endocrinologist

**Q3. Where do you practice?**

- Hospital
- Private practice
- Both

**Q4A. In which department of France do you work?** *[drop-down list; only one choice possible; for respondents in FRANCE]*

**Q4B. In which region do you work?** *[drop-down list; only one choice possible; for respondents in BELGIUM]*

1. **THE 1^st^ CONSULTATION**

**Q5. In your center, how long does it take to get a first appointment?** *[only one choice possible]*

- Less than a month
- 1 to 3 months
- More than 3 months

**Q6. According to your patients’ feedback and on a scale of 1 to 5, what criteria do they use to choose the MAR center for their first fertility consultation?** *[1 = criterion not very important; 5 = criterion very important]*

|  | 1 | 2 | 3 | 4 | 5 |
| --- | --- | --- | --- | --- | --- |
| Close location |  |  |  |  |  |
| The center’s reputation (success rate, number of cycles completed, specific healthcare professionals, etc.) |  |  |  |  |  |
| Preference for a private or public-sector center |  |  |  |  |  |
| Referral by a friend or patient |  |  |  |  |  |
| Referral by a healthcare professional |  |  |  |  |  |
| Social networks, blogs, media, or the internet |  |  |  |  |  |
| Waiting time for an appointment |  |  |  |  |  |
| Media coverage of certain centers |  |  |  |  |  |
| The possibility to participate in a study for eligible patients |  |  |  |  |  |

**Q7. How do you take into account lifestyles (dietary and/or health practices) and screening for toxic factors (occupational or personal exposure) before initiating MAR treatment?** *[multiple answers possible]*

- Written questionnaire
- Oral questionnaire
- Biological check-up
- I do not take these factors into account

**Q8. At this first appointment, what information about the MAR treatment pathway do you give your patients?** *[multiple answers possible]*

- I provide documentation (brochures, flyers, videos, websites, etc.)
- I recommend getting in touch with patient associations
- I recommend an informational meeting on therapeutic education, organized by the center
- I inform patients of the possibility of meeting a psychologist at the center
- Nothing at the first appointment; I wait for later consultations before making recommendations

1. **SUPPORT AT INITIATION AND DURING TREATMENT**

**Q9. What do you do if patients have trouble understanding treatment instructions?** *[multiple answers possible]*

- I take the time to re-explain (diagrams, drawings, etc.)
- I refer the patient to telemedicine appointments
- I ask patients to bring a trusted person with them to rephrase and simplify what I say
- I refer the patient to a nurse/midwife to explain the treatment
- I use a translator
- Other [please specify] _________________________________________________________

**Q10. How do you ensure that injections are carried out correctly at your center?**

|  | Always | Sometimes | Rarely | Never |
| --- | --- | --- | --- | --- |
| I personally demonstrate how to perform the injections correctly |  |  |  |  |
| Injection training is provided by the center’s (para)medical team |  |  |  |  |
| I provide digital tools (tutorials, videos, apps) to empower the couple/partner as much as possible |  |  |  |  |
| I propose a program to support the patient’s pathway (toll-free number, membership in a patient support group) |  |  |  |  |

**Q11. In your opinion, on a scale of 1 to 5, how frequent are the types of treatment errors listed below?** *[1 = infrequent; 5 = very frequent]*

|  | 1 | 2 | 3 | 4 | 5 |
| --- | --- | --- | --- | --- | --- |
| Dosage and/or handling errors |  |  |  |  |  |
| Forgetting to take treatment |  |  |  |  |  |
| Storage problems |  |  |  |  |  |
| Wrong time of medication intake |  |  |  |  |  |
| Inverting the different drugs |  |  |  |  |  |
| Missing a follow-up/stimulation monitoring appointment |  |  |  |  |  |
| Nurse injection error |  |  |  |  |  |
| Incorrect dispensing of medication (missing product, prescription errors, etc.) |  |  |  |  |  |
| Errors caused by the center |  |  |  |  |  |

1. **MANAGING FAILURE**

**Q12. What procedures have you set up to manage these two types of failure?** *[multiple answers possible]*

|  | Failure before transfer | Failure after embryo transfer |
| --- | --- | --- |
| Training of the team in announcing and managing failure |  |  |
| An appointment is always scheduled with the patient and partner (if applicable) |  |  |
| An additional check-up is offered for the patient and her partner (if applicable) |  |  |
| Collaboration with patients’ support groups has been proposed |  |  |
| Psychological support is available |  |  |
| Alternative medicine workshops are made available (acupuncture, hypnosis, yoga, etc.) |  |  |
| Participation in discussion groups is offered |  |  |
| None |  |  |
| Other [please specify] |  |  |

1. **TRENDS IN MAP FOLLOWING THE REVISED FRENCH BIOETHICS LAW AND NEW PATIENT RIGHTS** *[title for French respondents]*

**5. TRENDS IN MAR** *[title for Belgian respondents]*

**Q13. In your opinion, on a scale of 1 to 5, what is your patients’ level of knowledge about the following aspects of MAR treatment?** *[1 = low level of knowledge; 5 = very good level of knowledge]*

|  | 1 | 2 | 3 | 4 | 5 |
| --- | --- | --- | --- | --- | --- |
| Prerequisites for access to MAP (infertile couples, lesbian couples and single women) |  |  |  |  |  |
| Fertility preservation (gamete preservation) |  |  |  |  |  |
| Embryo preservation |  |  |  |  |  |
| Patient rights (excused absences from work) |  |  |  |  |  |
| Administrative procedures associated with the treatment (consent, legal parenthood, etc.) |  |  |  |  |  |
| Reimbursement conditions for protocols (age limits, number of protocols, etc.) |  |  |  |  |  |

**Q14. What are the main obstacles currently encountered in your activity?** *[multiple choices possible]*

- A very large number of active patients
- New applications (following the 2021 bioethics law) *[French only]*
- Lack of medical staff
- Lack of paramedical staff
- Waiting times too long for the first appointment
- Waiting times too long between the first appointment and initiation of treatment
- No preimplantation diagnosis *[French only]*
- Lack of financial resources and equipment

**Q15. How has your practice changed since the revision of the bioethics law (single women and lesbian couples)?** *[several choices possible] [For FRENCH respondents only]*

- Setting up special appointments for new patient profiles
- Recruiting healthcare professionals
- Recruitment of administrative staff
- Implementation of follow-up tools for ovarian stimulation via mobile apps or other means
- My practice hasn’t changed
